# Assessing the quality of ChatGPT's responses to questions related to radiofrequency ablation for varicose veins

**DOI:** 10.1016/j.jvsv.2024.101985

**Published:** 2024-09-25

**Authors:** Muhammad Anees, Fareed Ahmed Shaikh, Hafsah Shaikh, Nadeem Ahmed Siddiqui, Zia Ur Rehman

**Affiliations:** a Section of Vascular Surgery, Department of Surgery, Aga Khan University Hospital, Karachi, Pakistan; b Liaquat National Medical College, Karachi, Pakistan

**Keywords:** Artificial intelligence, ChatGPT, Large language model, Radiofrequency ablation, Varicose veins

## Abstract

**Objective:**

This study aimed to evaluate the accuracy and reproducibility of information provided by ChatGPT, in response to frequently asked questions about radiofrequency ablation (RFA) for varicose veins.

**Methods:**

This cross-sectional study was conducted at The Aga Khan University Hospital, Karachi, Pakistan. A set of 18 frequently asked questions regarding RFA for varicose veins were compiled from credible online sources and presented to ChatGPT twice, separately, using the new chat option. Twelve experienced vascular surgeons (with >2 years of experience and ≥20 RFA procedures performed annually) independently evaluated the accuracy of the responses using a 4-point Likert scale and assessed their reproducibility.

**Results:**

Most evaluators were males (n = 10/12 [83.3%]) with an average of 12.3 ± 6.2 years of experience as a vascular surgeon. Six evaluators (50%) were from the UK followed by three from Saudi Arabia (25.0%), two from Pakistan (16.7%), and one from the United States (8.3%). Among the 216 accuracy grades, most of the evaluators graded the responses as comprehensive (n = 87/216 [40.3%]) or accurate but insufficient (n = 70/216 [32.4%]), whereas only 17.1% (n = 37/216) were graded as a mixture of both accurate and inaccurate information and 10.8% (n = 22/216) as entirely inaccurate. Overall, 89.8% of the responses (n = 194/216) were deemed reproducible. Of the total responses, 70.4% (n = 152/216) were classified as good quality and reproducible. The remaining responses were poor quality with 19.4% reproducible (n = 42/216) and 10.2% nonreproducible (n = 22/216). There was nonsignificant inter-rater disagreement among the vascular surgeons for overall responses (Fleiss' kappa, −0.028; *P* = .131).

**Conclusions:**

ChatGPT provided generally accurate and reproducible information on RFA for varicose veins; however, variability in response quality and limited inter-rater reliability highlight the need for further improvements. Although it has the potential to enhance patient education and support healthcare decision-making, improvements in its training, validation, transparency, and mechanisms to address inaccurate or incomplete information are essential.

**Article Highlights**

• **Type of Research:** Single-center cross-sectional study
• **Key Findings:** A total of 12 vascular surgeons rated the responses by ChatGPT to frequently asked questions as mostly accurate (72.7%). Overall, 89.8% of the responses were reproducible. There was no agreement among the vascular surgeons for overall responses (Fleiss' kappa: −0.028; *P* = .131).
• **Take Home Message:** ChatGPT provided generally accurate and reproducible information with limited reliability for patients seeking information on radiofrequency ablation for varicose veins.

ChatGPT, an artificial intelligence (AI)-based large language model (LLM) developed by OpenAI, has transformed how individuals access health information online.^1^ Capable of generating human-like text, LLMs have the potential to improve healthcare communication by providing rapid responses in a user-friendly manner to patient inquiries.^2^^,^^3^ Unlike traditional search engines, which may lack personalization, LLMs overcome communication barriers^4^ and provide a centralized and intuitive approach to address patient queries,^5^ reflecting their increasing popularity for health-related questions.^6^^,^^7^ This factor is particularly important as more individuals turn to the internet for health information.^8^ A study in 2020 showed that more than one-half of Europeans (55%) searched for health information online.^9^ Similarly, in the United States, the percentage of people seeking online health information increased from 62.8% in 2008 to 74.7% in 2017.^10^ This finding highlights the increasing importance of evaluating LLMs to ensure they provide accurate and reliable health information.

Despite the convenience and accessibility of LLMs, concerns remain regarding the quality of AI-generated health information. This point is particularly important for conditions like varicose veins, which affect a significant portion of the population worldwide.^11^ Varicose veins are a common vascular disorder, with prevalences ranging from 2% to 73% globally.^11^ In the United States alone, it ranks among the top 10 reasons for medical consultations and affects 23% of adults.^12^^,^^13^ A study by Harsha et al^14^ showed a consistent increase in online search queries related to varicose vein treatment between 2004 and 2012, underscoring the demand for reliable information. Given that online health information affects 70% of patient's healthcare decisions, assessing the accuracy of AI-generated health information is essential.^10^

This study aimed to evaluate the accuracy and reproducibility of the information provided by a popular LLM in response to frequently asked questions (FAQs) regarding radiofrequency ablation (RFA) for varicose veins. RFA is a minimally invasive procedure frequently used in the treatment of varicose veins.^15^ It is considered highly effective, offering significant therapeutic benefits over traditional methods,^16^ such as improving the quality of life, enabling early resumption of daily activities,^17^ minimizing the likelihood of recurrence,^18^ and decreasing resource use.^19^ By examining the quality of information provided by ChatGPT, we sought to address a critical gap in understanding the accuracy of AI-generated health information.

## Methods

This cross-sectional study was conducted in the section of Vascular Surgery at The Aga Khan University, Karachi, Pakistan. The Ethics and Review Committee of The Aga Khan University (2023-9386-26,953) deemed the study exempt.

Initially, we identified 39 FAQs regarding RFA for varicose veins from reputable sources, including UCLA Health, Provascular MD, Hamilton Vascular, and Varicose Veins Doctors New York.^20^, ^21^, ^22^, ^23^ Two additional questions were contributed by experienced vascular surgeons, bringing the total to 41 questions. We excluded 23 questions with synonymous meanings, ambiguous interpretations, or those that could vary among individuals. To enhance precision and ensure relevant responses from ChatGPT, we modified questions that focused solely on RFA by adding the term varicose veins. For example, “How long does the radiofrequency ablation procedure take?” was revised to “How long does the radiofrequency ablation procedure take to treat varicose veins?“ This process resulted in a final set of 18 questions, including questions related to pretreatment concerns, procedural information, recovery, and outcomes (Supplemental Table).

We used ChatGPT version 3.5 to generate responses to the final set of questions in September 2023. Each question was entered twice on two separate occasions, one week apart, using the new chat feature to obtain two responses per question. This was done to assess the reproducibility of responses to each question. To simulate real-world scenarios where patients seek rapid access to easily understandable information, we used the prompt, “Give a concise response,” before administering the questions to ChatGPT.

Twelve experienced vascular surgeons independently evaluated the accuracy and reproducibility of the ChatGPT responses. Inclusion criteria for vascular surgeons included performing ≥20 RFAs for varicose veins annually and having a minimum of 2 years of experience. Invitations were sent via emails to evaluators across tertiary care hospitals in Pakistan and internationally (United Kingdom, United States, and Saudi Arabia) using convenience sampling. Those who agreed to participate were sent a secure Google Forms link. Participation in the survey was voluntary and anonymous, with informed consent obtained from all participants.

The vascular surgeons assessed the accuracy of ChatGPT's responses using a 4-point Likert scale.

1. Entirely inaccurate.
2. A mixture of accurate and inaccurate information.
3. Accurate but insufficient: All information provided is accurate but lacks comprehensiveness. An experienced vascular surgeon could add more details if questioned by a patient.
4. Comprehensive: This entails being both accurate and complete, providing all the information an experienced vascular surgeon might offer if posed this question by a patient.^24^

Additionally, each evaluator independently assessed reproducibility by comparing the similarity of the two responses for each question. For questions with differing responses, such as conflicting information or differences in the level of detail, both versions were assessed separately by the evaluators.

We performed data analysis on SPSS version 28 (IBM SPSS Inc., Armonk, NY). Continuous variables are presented as mean ± standard deviation and the responses to each question based on the Likert scale are presented as frequency and percentages. We assessed interrater reliability using Fleiss' Kappa statistics along with its 95% confidence interval. According to Landis and Koch, a kappa value of <0.00 indicates poor agreement, 0.00 to 0.20 suggests slight agreement, 0.21 to 0.40 indicates fair agreement, 0.41 to 0.60 reflects moderate agreement, 0.61 to 0.80 indicates substantial agreement, and 0.81 to 1.00 indicates almost perfect agreement.^25^ A *P* value of <.05 was considered statistically significant. We used the approach used by Yeo et al^24^ to assess the reproducibility of the responses provided by ChatGPT. Responses were divided into two sets to assess reproducibility: grades 1 and 2 formed the first set and grades 3 and 4 formed the second. Responses falling into different categories were considered nonreproducible. Additionally, we categorized responses based on the quality of information provided as good quality if they received a grade of ≥3 and poor quality if they received a grade of ≤2.

## Results

We received a total of 15 responses, of which 3 were excluded because they did not fulfill the inclusion criteria. Therefore, we included 12 vascular surgeons in our analysis, predominantly male (83.3%), with a mean of 12.3 ± 6.2 years of experience. Most evaluators were based in the UK (50%), followed by Saudi Arabia (25%), Pakistan (16.7%), and the United States (8.3%) (Table I).

**Table I:** Demographics of the vascular surgeons

Characteristic	Value
Age of vascular surgeons, years	47.1 ± 5.9
Gender	
Male	10 (83.3)
Female	2 (16.7)
Years of experience	12.3 ± 6.2
No. of RFA performed annually	
20-40	6 (50.0)
>40	6 (50.0)
Country	
UK	6 (50.0)
Pakistan	2 (16.7)
Saudi Arabia	3 (25.0)
United States	1 (8.3)

*RFA*, Radiofrequency ablation.

Values are mean ± standard deviation or number (%).

Of 18 questions, 12 responses were rated as either accurate but insufficient or comprehensive by ≥75% (9/12) of the evaluators (Table II). Commonly assigned grades for accuracy were comprehensive or accurate but insufficient for most of the responses (n = 17/18 [94.4%]). Among the total 216 accuracy grades, the most frequently assigned grades were comprehensive (n = 87/216 [40.3%]) and accurate but insufficient (n = 70/216 [32.4%]), whereas only 17.1% (n = 37/216) were graded as a mixture of accurate and inaccurate information and 10.8% (n = 22/216) as entirely inaccurate. Accuracy grades assigned to each question are summarized in the Figure.

**Table II:** Accuracy grades and reproducibility assessed by the vascular surgeons

Questions	Entirely inaccurate	A mixture of accurate and inaccurate information	Accurate but insufficient	Comprehensive	Reproducibility
1	0 (0)	4 (33.3)	4 (33.3)	4 (33.3)	12 (100.0)
2	1 (8.3)	2 (16.7)	4 (33.3)	5 (41.7)	12 (100.0)
3	1 (8.3)	1 (8.3)	5 (41.7)	5 (41.7)	11 (91.7)
4	1 (8.3)	1 (8.3)	5 (41.7)	5 (41.7)	12 (100.0)
5	1 (8.3)	2 (16.7)	3 (25.0)	6 (50.0)	12 (100.0)
6	1 (8.3)	2 (16.7)	2 (16.7)	7 (58.3)	11 (91.7)
7	1 (8.3)	3 (25.0)	1 (8.3)	7 (58.3)	10 (83.3)
8	1 (8.3)	2 (16.7)	5 (41.7)	4 (33.3)	12 (100.0)
9	1 (8.3)	3 (25.0)	3 (25.0)	5 (41.7)	10 (83.3)
10	1 (8.3)	4 (33.3)	4 (33.3)	3 (25.0)	8 (66.7)
11	2 (16.7)	1 (8.3)	4 (33.3)	5 (41.7)	12 (100.0)
12	2 (16.7)	0 (0)	5 (41.7)	5 (41.7)	12 (100.0)
13	1 (8.3)	2 (16.7)	6 (50.0)	3 (25.0)	12 (100.0)
14	1 (8.3)	3 (25.0)	5 (41.7)	3 (25.0)	9 (75.0)
15	3 (25.0)	4 (33.3)	1 (8.3)	4 (33.3)	7 (58.3)
16	2 (16.7)	1 (8.3)	3 (25.0)	6 (50.0)	8 (66.7)
17	1 (8.3)	0 (0)	7 (58.3)	4 (33.3)	12 (100)
18	1 (8.3)	2 (16.7)	3 (25.0)	6 (50.0)	12 (100)
Total	22 (10.8)	37 (17.1)	70 (32.4)	87 (40.3)	194 (89.8)

Values are number (%).

**Figure:**
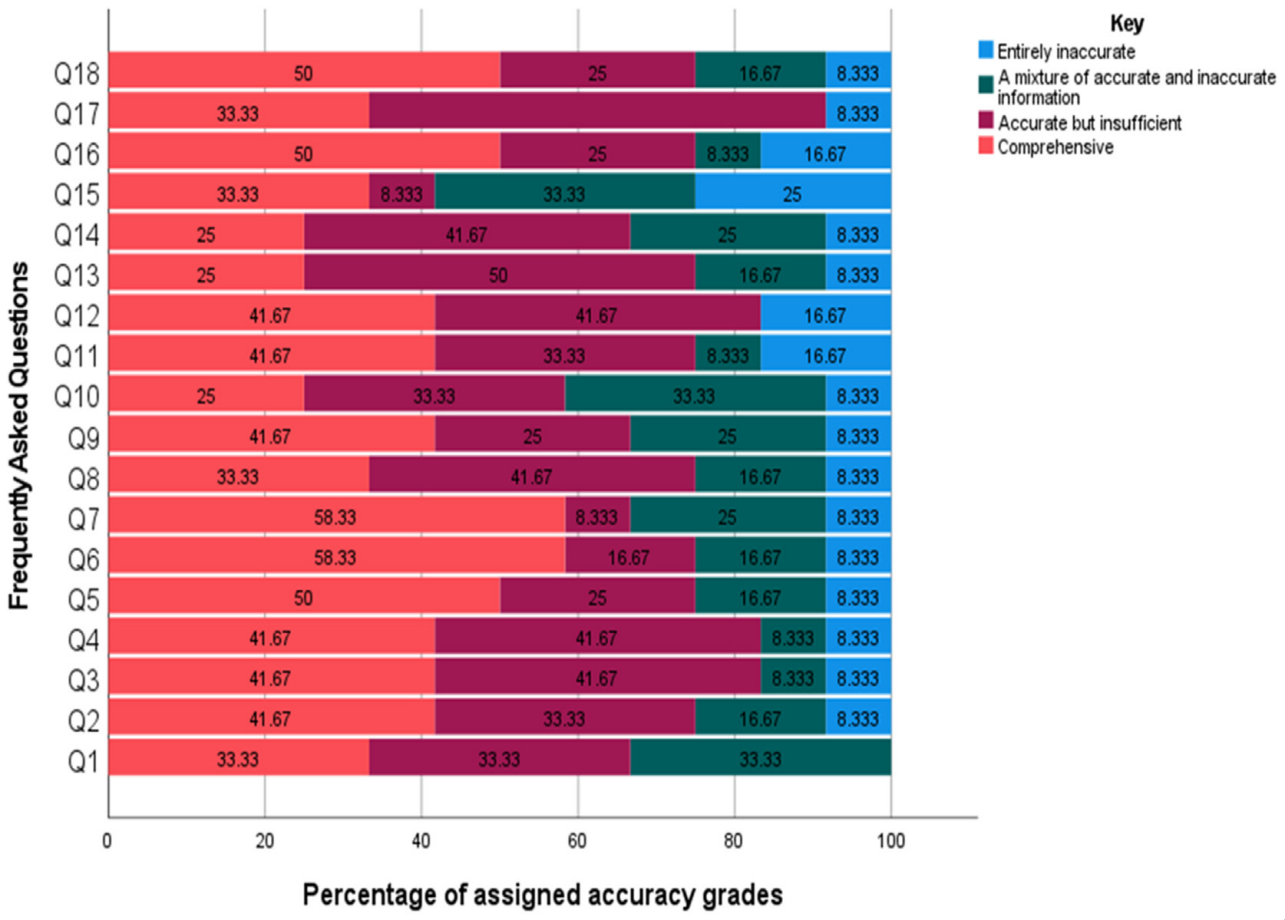
Clustered bar graph representing the percentage of assigned accuracy grades to each frequently asked question (FAQ).

The reproducibility of the responses was high, with 89.8% of the responses (n = 194/216) being consistent between the two entries (Table II). Questions 1, 2, 4, 5, 8, 11, 12, 13, and 18 achieved 100% reproducibility.

Table III summarizes the mean grades and inter-rater reliability among the 12 evaluators for all the questions divided into 3 categories: pretreatment concerns, procedural information, recovery, and outcomes. The mean accuracy grades were 3.08 ± 0.07 for pretreatment concerns, 3.00 ± 0.28 for procedural information, and 3.00 ± 0.16 for recovery and outcomes. Overall, the mean grade was 3.03 ± 0.19 and there was nonsignificant disagreement among the evaluators (Fleiss' Kappa, −0.028; 95% confidence interval, −0.063 to 0.008; *P* = .131).

**Table III:** Overall mean and inter-rater reliability between the 12 vascular surgeons for frequently asked questions (FAQs) with a 95% confidence interval

Questions	Mean ± standard deviation	Fleiss’ kappa	95% Confidence interval	*P* value
Pretreatment concerns (1, 2, 3, 4, 11, 12)	3.08 ± 0.07	−0.063	−0.128 to 0.001	.054
Procedural Information (5, 6, 14, 15, 16, 17)	3.00 ± 0.28	−0.002	−0.063 to 0.059	.942
Recovery and Outcomes (7, 8, 9, 10, 13, 18)	3.00 ± 0.16	−0.033	−0.095 to 0.029	.295
Overall	3.03 ± 0.19	−0.028	−0.063 to 0.008	.131

Among the total responses, 70.4% (152/216) were identified as being of good quality and reproducible, with none falling under the category of good quality and nonreproducible (Table IV). A total of 19.4% responses (42/216) were deemed poor quality and reproducible compared with 10.2% (22/216) identified as poor quality and nonreproducible.

**Table IV:** Quality and reproducibility of the responses as evaluated by vascular surgeons

Questions	Good quality, reproducible	Good quality, nonreproducible	Poor quality, nonreproducible	Poor quality, reproducible
1	8 (66.7)	0 (0.0)	0 (0.0)	4 (33.3)
2	9 (75.0)	0 (0.0)	0 (0.0)	3 (25.0)
3	9 (75.0)	0 (0.0)	1 (8.3)	2 (16.7)
4	10 (83.3)	0 (0.0)	0 (0.0)	2 (16.7)
5	9 (75.0)	0 (0.0)	0 (0.0)	3 (25.0)
6	9 (75.0)	0 (0.0)	1 (8.3)	2 (16.7)
7	8 (66.7)	0 (0.0)	2 (16.7)	2 (16.7)
8	9 (75.0)	0 (0.0)	0 (0.0)	3 (25.0)
9	7 (58.3)	0 (0.0)	2 (16.7)	3 (25.0)
10	6 (50.0)	0 (0.0)	4 (33.3)	2 (16.7)
11	9 (75.0)	0 (0.0)	0 (0.0)	3 (25.0)
12	10 (83.3)	0 (0.0)	0 (0.0)	2 (16.7)
13	9 (75.0)	0 (0.0)	0 (0.0)	3 (25.0)
14	8 (66.7)	0 (0.0)	3 (25.0)	1 (8.3)
15	5 (41.7)	0 (0.0)	5 (41.7)	2 (16.7)
16	7 (58.3)	0 (0.0)	4 (33.3)	1 (8.3)
17	11 (91.7)	0 (0.0)	0 (0.0)	1 (8.3)
18	9 (75.0)	0 (0.0)	0 (0.0)	3 (25.0)

Values are number (%).

## Discussion

This study evaluated the accuracy and reproducibility of ChatGPT's responses to FAQs by patients regarding RFA for varicose veins. Overall, the majority of responses provided by ChatGPT were accurate and showed high reproducibility. ChatGPT performed consistently across different subcategories of questions, including pretreatment concerns, procedural information, recovery, and outcomes. However, there was no level of agreement among the evaluators regarding the accuracy of ChatGPT's responses. Our study highlights the potential of using generative AI in enhancing patient education, health care decision-making, and thus outcomes.

There is a notable increase in online activity seeking health information,^26^ as demonstrated by Harsha et al's findings^14^ on the increasing trend of searches related to varicose vein treatment. This increase in online activity raises concern regarding health misinformation, which can impact patient decision-making adversely and can lead to harmful outcomes,^27^ including increased fear and anxiety and decreased access to health care.^28^ Previous studies by Yan et al^29^ and Ching et al^30^ highlight the inconsistent quality and poor readability of online information available on the internet to patients regarding varicose veins. These challenges present an opportunity for LLMs to offer promising new avenues for patients seeking information on varicose veins and RFA. Unlike traditional search engines, LLMs provide concise and well-summarized information, which can aid in making informed decisions.

Recent literature has placed significant emphasis on the use of ChatGPT for enhancing patient education across multiple medical specialties; however, there are limited studies on its role in the field of vascular surgery. Studies have evaluated its accuracy in responding to patient queries across diverse medical topics, including cancer treatment,^24^^,^^31^^,^^32^ surgical procedures,^33^^,^^34^ and various medical conditions.^35^, ^36^, ^37^ The prevailing consensus from these studies suggests that AI-powered chatbots hold significant promise for improving patient education and overall health outcomes. A study by Athavale et al^38^ investigated the potential of chatbots, specifically ChatGPT versions 3.5 and 4.0, in the management of chronic venous disease, focusing on both administrative and complex medical inquiries. Their findings indicated superior performance by version 4.0 on both categories of queries. Notably, their evaluation assigned a grade of 1 (indicating appropriate and complete) to 9 of 20 complex medical queries answered by version 3.5 and 15 of 20 by version 4.0.^38^ Our findings align with these observations, with comprehensive being the most frequently assigned grade followed by accurate but insufficient. However, our study used a larger pool of independent evaluators—12 compared with the 2 used by Athavale et al—and we only used the cost-free, more accessible version (3.5) of ChatGPT.

Our study reports an overall reproducibility of 89.8% for responses by ChatGPT. This result aligns with findings from previous studies investigating response reproducibility across various topics, including osteoporosis (86.1%),^39^ bariatric surgery (90.7%),^40^ cirrhosis and hepatocellular carcinoma (90.5%),^24^ and head and neck cancers (94.1%).^41^ Additionally, although ChatGPT generated primarily good quality responses, our study revealed that 29.6% of its answers were classified as poor quality, highlighting its limitations in providing high-quality information consistently. LLMs can sometimes hallucinate, generating information that seems to be accurate but is factually incorrect. This underscores the need for cautious use of LLMs and emphasizes the importance of further improvements to enhance information quality. One significant concern with LLMs is their potential to produce information without critically evaluating its value or relevance. Although ChatGPT is designed to mimic human-like conversation, it cannot prioritize or assess the accuracy of the information it generates.^42^ As a result, it may disseminate outdated or irrelevant information inadvertently. This poses risks to patient education and decision-making, because patients might receive incorrect or misleading information that could impact their understanding and choices regarding health care.^43^ As LLMs continue to evolve, it is crucial to develop mechanisms for validating and updating training datasets to ensure that the generated information is both current and accurate. To improve reliability, developers should consider establishing partnerships with publishers to access scientific literature for training purposes. Currently, ChatGPT provides information without referencing its sources, and its reliance on undisclosed, freely available data diminishes the reliability of its information.^43^ Increasing transparency about how information is sourced and verified could build trust in LLMs for patient education. Generative chatbots that can reference their sources or provide links to further reading might be more reliable and useful for patients and professionals.

Despite most responses being graded as accurate, there was poor agreement among the evaluators regarding information provided by ChatGPT. Compared with previous studies assessing inter-rater agreement among evaluators for grading the information provided by ChatGPT, our study shows a lower level of agreement. A study by Draschl et al^44^ on evaluating the performance of ChatGPT in answering complex orthopedic questions demonstrated diverse levels of inter-rater agreement. Another study by Walker et al^45^ demonstrated substantial agreement among evaluators assessing the reliability of the information provided by ChatGPT on conditions including gallstone disease, pancreatitis, liver cirrhosis, pancreatic cancer, and hepatocellular carcinoma. A key difference in our study was the inclusion of more evaluators compared with the three evaluators in previous studies. Factors contributing to poor agreement among evaluators in our study may include personal biases and subjective interpretation of the 4-point Likert scale.^46^ The limited inter-rater reliability underscores the need for standardized and objective tools in future studies to assess the accuracy and reliability of AI-generated information. Although most responses were graded as accurate, poor agreement among evaluators indicates that caution should be exercised when interpreting the study's results.

This study comprehensively assessed the accuracy of information provided by ChatGPT on RFA for varicose veins. We used FAQs from well-known institutions that underwent a screening process to provide a comprehensive and representative sample of patient queries. Additionally, the provided information was independently assessed by 12 experienced vascular surgeons across different countries to adequately assess the accuracy, reproducibility, and inter-rater reliability of the responses.

Our study has several limitations. Although a total of 40% of the responses were graded as comprehensive, a few responses contained inaccurate information, posing potential risks to patients who seek information from ChatGPT without guidance from a healthcare provider. This result suggests that ChatGPT can potentially complement patient care rather than replace the need for licensed healthcare provider assistance. Because ChatGPT is anticipated to undergo ongoing enhancements, both the quality and consistency of responses are expected to improve over time. Second, although we assessed a substantial number of questions from reputable institutions, it is possible that the selected queries did not cover all the relevant patient inquiries concerning RFA for varicose veins. Third, given that the experts were aware that ChatGPT generated these responses, they may have applied a more stringent grading approach, potentially leading to an underestimation of ChatGPT's performance. Fourth, although we used a 4-point Likert scale to evaluate response accuracy objectively, there exists a potential for subjective interpretation of the scale by evaluators leading to limited agreement. Thus, it would have been prudent to pilot test or validate the scale to ensure its reliability and validity. Fifth, the database used to train ChatGPT is limited to information available until 2021. Consequently, some topics may contain outdated information, potentially resulting in inaccuracies in responses.

## Conclusions

ChatGPT generally provides accurate and reproducible information for patients seeking information on RFA for varicose veins. However, the study also highlights variability in the quality of responses and evaluator agreement, highlighting the need for caution in relying on AI-generated health information. The findings suggest that LLMs have the potential to enhance patient education and support informed decision-making by providing accessible and timely information. Nevertheless, the variability in response quality and evaluator agreement points to the necessity for continuous improvements in AI training and validation processes. Future developments should focus on integrating mechanisms for validating and updating information, increasing transparency about data sources, and addressing limitations in response accuracy.

## Author Contributions

Conception and design: MA, FS, HS, NS, ZR

Analysis and interpretation: MA

Data collection: FS

Writing the article: MA, FS, HS

Critical revision of the article: MA, FS, NS, ZR

Final approval of the article: MA, FS, HS, NS, ZR.

Statistical analysis: MA

Obtained funding: Not applicable

Overall responsibility: FS

MA and FS contributed equally to this article and share co-first authorship.

## Funding

None.

## Disclosures

None.
