# Supplementary material for: Assessing the quality of ChatGPT's responses to questions related to radiofrequency ablation for varicose veins
Source: J Vasc Surg Venous Lymphat Disord. 2024 Sep 25;13(1):101985. doi: 10.1016/j.jvsv.2024.101985 (PMC11764857; doi:10.1016/j.jvsv.2024.101985)
Supplement: Supplementary File [file mmc1.docx]

Supplementary File: Frequently Asked Questions and ChatGPT Responses

| Question | ChatGPT Response 1 | ChatGPT Response 2 |
| --- | --- | --- |
| 1. What is Radiofrequency Ablation for varicose veins? | Radiofrequency ablation (RFA) for varicose veins is a minimally invasive procedure that uses radiofrequency energy to heat and close off problematic veins. This helps improve blood flow and alleviate symptoms associated with varicose veins. | Similar |
| 2. Why do you treat varicose veins with Radiofrequency Ablation? | Radiofrequency Ablation (RFA) is used to treat varicose veins because it is a minimally invasive procedure that uses radiofrequency energy to heat and close the affected veins. This helps to alleviate symptoms and improve the appearance of varicose veins. RFA is effective in sealing the problematic veins, redirecting blood flow to healthier vessels, and reducing the likelihood of complications. | Similar |
| 3. How is Radiofrequency Ablation different from open surgery for varicose veins? | Radiofrequency ablation (RFA) for varicose veins is a minimally invasive procedure that uses radiofrequency energy to heat and close off the affected veins. It involves inserting a catheter into the vein, which delivers heat and causes the vein to collapse. This method is less invasive than open surgery. | Radiofrequency ablation (RFA) and laser ablation are both minimally invasive treatments for varicose veins, but they use different sources of energy.  Radiofrequency Ablation (RFA):  Uses radiofrequency energy to heat and close the affected vein.  Involves the insertion of a catheter into the vein, which emits radiofrequency waves to seal the vein walls.  Generally, causes less discomfort and bruising compared to traditional vein-stripping surgery.  Laser Ablation:  Involves using laser energy to heat and close the varicose vein.  Similar to RFA, a catheter is inserted into the vein, and laser energy is delivered to seal the vein walls.  Can be an effective alternative to surgical procedures with reduced recovery time.  Both RFA and laser ablation aim to redirect blood flow to healthier veins and improve circulation. The choice between the two may depend on factors such as the physician's expertise, patient preference, and specific characteristics of the varicose veins being treated. |
| 4. Which of the two options, Radiofrequency Ablation, or open surgery, is better for treating varicose veins? | The choice between Radiofrequency Ablation and open surgery for treating varicose veins depends on the specific characteristics of the patient's condition. Both methods are effective, but less invasive procedures like Radiofrequency Ablation are often preferred for their quicker recovery times and lower risk of complications. However, the best approach should be determined through consultation with a healthcare professional based on individual circumstances. | The choice between Radiofrequency Ablation (RFA) and open surgery for treating varicose veins depends on individual patient factors. Generally, RFA is considered less invasive and has a shorter recovery time compared to open surgery. However, the best option varies based on factors such as the size and location of the varicose veins, overall health, and patient preferences. Consult with a vascular specialist to determine the most suitable treatment for your specific case. |
| 5. Is Radiofrequency Ablation painful? | Radiofrequency ablation (RFA) is generally considered a minimally invasive procedure and is often well-tolerated by patients. Local anesthesia is typically used to numb the area, which can reduce pain during the procedure. However, some patients may still experience discomfort or mild pain. After the procedure, individuals may have some soreness or discomfort at the treatment site, but this is usually temporary. Overall, the level of pain can vary among individuals, and it's advisable to discuss any concerns with your healthcare provider. | Similar |
| 6. How long does the Radiofrequency Ablation procedure take to treat varicose veins? | The Radiofrequency Ablation (RFA) procedure for treating varicose veins usually takes about 30 minutes to an hour. | The Radiofrequency Ablation (RFA) procedure for treating varicose veins typically takes about 45 minutes to an hour |
| 7. How quickly after Radiofrequency Ablation can I return to daily activities? | Recovery time after Radiofrequency Ablation varies, but many people can resume daily activities within a day or two. It's advisable to follow your healthcare provider's guidance based on your specific situation. | Recovery time after Radiofrequency Ablation (RFA) varies, but many people can return to daily activities within a day or two. However, it's essential to follow your doctor's recommendations and avoid strenuous activities for a short period after the procedure. |
| 8. How soon after Radiofrequency Ablation for varicose veins will my symptoms improve? | Improvement in symptoms after Radiofrequency Ablation for varicose veins is typically noticeable within a few days to weeks. However, individual responses may vary, and it's advisable to follow your healthcare provider's guidance for a more personalized timeline. | Similar |
| 9. What happens to the treated vein left behind in the leg after Radiofrequency Ablation? | After Radiofrequency Ablation (RFA) for varicose veins, the treated vein typically collapses and eventually gets reabsorbed by the body. Over time, the vein becomes fibrous tissue, and blood flow is rerouted to healthier veins. This minimally invasive procedure is designed to alleviate symptoms of venous insufficiency and improve blood circulation in the affected leg. | After Radiofrequency Ablation (RFA) for varicose veins, the treated vein typically collapses and is gradually absorbed by the body. Over time, the body reabsorbs the vein, and blood flow is rerouted through healthier veins. |
| 10. What potential risks and complications are associated with Radiofrequency Ablation for varicose veins? | Potential risks and complications of Radiofrequency Ablation (RFA) for varicose veins include infection, nerve injury, bruising, hematoma, and thermal skin burns. Rare but serious complications may involve deep vein thrombosis, pulmonary embolism, or damage to nearby blood vessels. It's essential to discuss individual risks with a healthcare provider before undergoing the procedure. | Potential risks and complications of Radiofrequency Ablation (RFA) for varicose veins may include:  Pain or Discomfort: Some patients may experience pain or discomfort during or after the procedure.  Bruising or Swelling: Bruising and swelling at the treatment site are common but usually temporary.  Skin Burns: In rare cases, skin burns may occur due to the heat generated during the procedure.  Nerve Injury: There is a small risk of damage to nearby nerves, which could result in numbness or tingling.  Blood Clots: Although rare, blood clots can form in the treated vein, potentially leading to more serious complications.  Infection: Infections at the catheter insertion site are possible but uncommon.  Allergic Reaction: Some patients may have an allergic reaction to the contrast dye used during the procedure.  Incomplete Closure: The treated vein may not close completely, requiring additional treatment.  Deep Vein Thrombosis (DVT): There is a slight risk of developing DVT, a blood clot in a deeper vein.  Recurrence of Varicose Veins: While RFA is effective, there is a chance of varicose veins recurring over time.  It's crucial to discuss these potential risks and complications with your healthcare provider before undergoing Radiofrequency Ablation. They can provide personalized information based on your medical history and assess whether the benefits of the procedure outweigh the risks in your specific case. |
| 11. Is Radiofrequency Ablation right for everyone for the treatment of varicose veins? | No, Radiofrequency Ablation (RFA) may not be suitable for everyone with varicose veins. The decision depends on various factors such as the size and location of the veins, overall health, and individual circumstances. Consultation with a healthcare professional is essential to determine the most appropriate treatment for a specific case. | Similar |
| 12. Will this (Radiofrequency Ablation) varicose vein surgery address all my vein problems? | Radiofrequency Ablation (RFA) is a minimally invasive procedure commonly used to treat varicose veins by closing them. However, it may not address all vein problems. The suitability of RFA depends on the specific characteristics of your veins and the underlying venous issues. Consult with a vascular specialist to determine the most appropriate treatment for your individual case. | Radiofrequency Ablation (RFA) is a minimally invasive procedure commonly used to treat varicose veins by closing them using heat generated by radiofrequency energy. While RFA is effective for many varicose veins, it may not address all types of vein problems. Other procedures, such as sclerotherapy or phlebectomy, might be considered depending on the nature and extent of your vein issues. It's essential to consult with a vascular specialist to determine the most suitable treatment for your specific condition. |
| 13. What can happen if varicose veins are left untreated? | If varicose veins are left untreated, complications may arise, including pain, swelling, skin changes, and in severe cases, ulcers or blood clots. It's advisable to consult a healthcare professional for proper evaluation and guidance on treatment options. | Similar |
| 14. Can I eat before my Radiofrequency Ablation procedure for the treatment of varicose veins? | Typically, it's advisable to follow fasting instructions provided by your healthcare provider before a Radiofrequency Ablation (RFA) procedure for varicose veins. This helps minimize potential complications during the intervention. Always consult with your healthcare team for specific guidance based on your individual case. | Typically, it's recommended to fast for a few hours before a Radiofrequency Ablation (RFA) procedure for varicose veins. However, the specific instructions may vary, so it's crucial to follow the guidelines provided by your healthcare provider. Always consult with your doctor for personalized advice based on your health and the details of your procedure. |
| 15. Will I be asleep for my Radiofrequency Ablation procedure for the treatment of varicose veins? | Yes, for a Radiofrequency Ablation (RFA) procedure for varicose veins, you are typically given local anesthesia or sedation to ensure you are comfortable and pain-free during the procedure. While you may be drowsy, you are not usually fully asleep. | Typically, Radiofrequency Ablation (RFA) for varicose veins is done under local anesthesia, so you may be awake but won't feel pain. The level of sedation can vary, and you should discuss this with your healthcare provider before the procedure. |
| 16. Can I drive after the Radiofrequency Ablation procedure for the treatment of varicose veins? | It's generally advisable to avoid driving immediately after a Radiofrequency Ablation (RFA) procedure for varicose veins, as the sedation or anesthesia used during the procedure may affect your ability to drive safely. It's important to follow your healthcare provider's recommendations and arrange for someone else to drive you home after the procedure. | In most cases, you can typically resume driving after Radiofrequency Ablation for varicose veins. However, it's crucial to follow your healthcare provider's advice, as individual recovery experiences may vary. Always consult with your doctor regarding any specific restrictions or recommendations based on your condition and the details of the procedure. |
| 17. Will I be sent home with bandages after Radiofrequency Ablation for the treatment of varicose veins? | Yes, bandages are typically applied after Radiofrequency Ablation for varicose veins, but the specific post-procedure care may vary. It's best to follow your healthcare provider's instructions. | Similar |
| 18. What happens if my leg starts to bleed or if my leg(s) start/continue hurting after Radiofrequency Ablation? | If you experience bleeding or persistent pain in your leg(s) after Radiofrequency Ablation (RFA), it's crucial to contact your healthcare provider immediately. These symptoms may indicate a complication or side effect that needs prompt medical attention. | Similar |
